# Spatial regulation of *expanded* transcription in the *Drosophila* wing imaginal disc

**DOI:** 10.1371/journal.pone.0201317

**Published:** 2018-07-31

**Authors:** Lan-Hsin Wang, Nicholas E. Baker

**Affiliations:** 1 Graduate Institute of Life Sciences, National Defense Medical Center, 161 Sec 6, Taipei, Taiwan; 2 Department of Genetics, Albert Einstein College of Medicine, Bronx, NY, United States of America; 3 Department of Developmental and Molecular Biology, Albert Einstein College of Medicine, Bronx, NY, United States of America; 4 Department of Ophthalmology and Visual Sciences, Albert Einstein College of Medicine, Bronx, NY, United States of America; Simon Fraser University, CANADA

## Abstract

Growth and patterning are coordinated during development to define organ size and shape. The growth, proliferation and differentiation of *Drosophila* wings are regulated by several conserved signaling pathways. Here, we show that the Salvador-Warts-Hippo (SWH) and Notch pathways converge on an enhancer in the *expanded* (*ex*) gene, which also responds to levels of the bHLH transcription factor Daughterless (Da). Separate cis-regulatory elements respond to Salvador-Warts-Hippo (SWH) and Notch pathways, to bHLH proteins, and to unidentified factors that repress *ex* transcription in the wing pouch and in the proneural region at the anterior wing margin. Senseless, a zinc-finger transcription factor acting in proneural regions, had a negative impact on *ex* transcription in the proneural region, but the transcriptional repressor Hairy had no effect. Our study suggests that a complex pattern of *ex* transcription results from integration of a uniform SWH signal with multiple other inputs, rather than from a pattern of SWH signaling.

## Introduction

One *Drosophila* gene that integrates signals from multiple growth pathways is *expanded* [1–4]. The *ex* gene encodes a FERM-domain protein that activates the Salvador-Warts-Hippo (SWH) pathway of tumor suppressors by at least two mechanisms. First, Ex directly binds with Yorkie (Yki), the *Drosophila* homolog of Yap and Taz, sequestering Yki in the cytoplasm [5,6]. Secondly, binding of Ex with another FERM domain-containing protein Schip1 can recruit the Hippo (Hpo) kinase Tao-1 and then phosphorylates Hpo [7]. Phosphorylation of Hpo, and then Warts, leads to the phosphorylation and cytoplasmic retention of Yki. Both mechanisms of cytoplasmic Yki retention prevent the expression of growth and survival genes including *cyclin E*, *diap1*, and *bantam* by this transcriptional coactivator [8–15].

**Fig 1:**
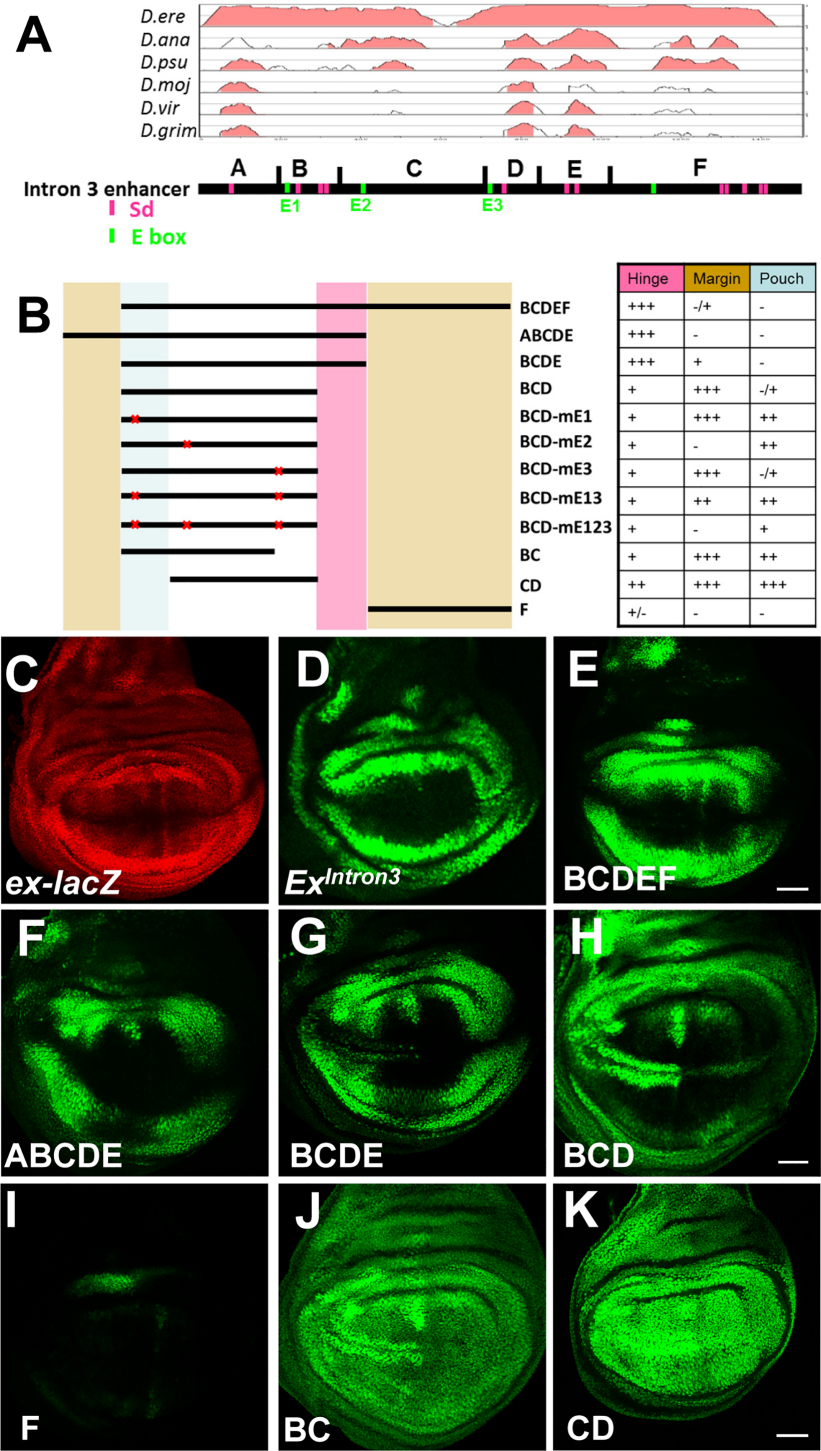
Analysis of the intron-3 enhancer. (A) Evolutionary conservation of the intron-3 enhancer. VISTA plot [41, 42] of the *D*. *melanogaster* intron-3 enhancer (Ex^Intron3^) aligned with sequence from *D*. *erecta*, *D*. *ananassae*, *D*. *pseudoobscura*, *D*. *mojavensis*, *D*. *virilis* and *D*. *grimshawl*. A window size of 100 bp is used, and regions that are greater than 70% identical are indicated in pink. Regions A-F are defined on the basis of the distribution of sequence conservation among *Drosophila*. ABCDEF is 1501 bp. A is 1–183; B is 184–345; C is 346–723; D is 724–842; E is 843–1010; F is 1011–1501. Putative E-box and Scalloped (Sd) binding sites are indicated. (B) Deletional or mutational constructs are generated as indicated. Spatial wing expression of each construct is summarized in the table. +++: strong expression; ++: middle expression; +: weak expression; +/-: much weaker or partial expression; -: no expression. Region directing expression in the hinge of wing disc is indicated in pink; regions directing expression in the neuronal precursors of wing disc are pointed in brown; region driving expression in the wing pouch is indicated in blue. (C-K) Wing expression patterns of *ex-LacZ* and the indicated genomic sub-fragments of Ex^Intron3^. Note the fragments BCDE, BCD, BC and CD drive GFP expression in neuronal precursors. Scale bars, 50 μm.

The *ex* gene is itself a transcriptional target of Yki and could behave as a negative feedback regulator of the SWH pathway [1]. Since feedback regulators are often among the most general transcriptional targets of signaling pathways, necessary in multiple contexts, they make good general reporters. Accordingly, the *ex-LacZ* enhancer trap is frequently used as a reporter of Yki activity. In the third instar wing disc, *ex-LacZ* is transcribed most highly in the proximal wing hinge primordium region of the wing disc, at lower levels in the wing pouch, and absent from the most proximal, wing margin primordium, suggesting a distal-to-proximal gradient of SWH activity that would allow Yki to promote most growth proximally [3,16] (Fig 1C). This is consistent with some models of wing disc growth, which suggest that proximal growth is predominantly under the control of Yki but that growth in the distal wing depends more on Dpp signaling [17,18], possibly because cells in the proximal wing are stretched in comparison to distal wing cells that are more compressed [19,20].

One factor potentially contributing to the gradient of Yki activity is Notch signaling, which is active at the wing margin [21]. Yki-dependent wing growth depends on Sd, Yki’s DNA-binding protein partner [16,22,23]. Notch induces expression of Vestigial, another Sd-binding protein that is proposed to compete with Yki for Sd and so limit Yki-induced growth in the distal wing pouch [3].

Besides Sd/Yki, under certain circumstances *ex* is transcriptionally regulated by the bHLH protein Daughterless (Da). In most cells, Da expression is held in check by its inhibitory heterodimer partner Emc, but if Emc is mutated Da expression upregulates and activates ex transcription, reducing growth and survival of *emc* mutant cells by activating the SWH pathway [4]. Unlike most cells, proneural regions, which are growth-arrested domains within which neural fate determination occurs, express Emc at low levels, which allows Da levels to rise and heterodimerize with the proneural bHLH proteins that drive neural fate determination and differentiation [24]. Unlike *emc* mutant cells, however, proneural cells do not seem to activate *ex* transcription, despite lower Emc and higher Da levels. For example the anterior wing margin contains a proneural region that gives rise to the innervated sensory bristles of the anterior wing margin of the adult fly [25,26]. The primordium of the anterior wing margin lies in the distal center of the wing disc, where *ex-LacZ* is lowest, despite elevated Da and reduced Emc levels there [24]. Another proneural region, the morphogenetic furrow of the eye imaginal disc, also exhibits low *ex-LacZ* levels despite also lacking Emc protein and expressing elevated Da [1,24].

Through our analysis of *ex* regulation by Da, we identified an enhancer within intron 3 that replicates most features of *ex* transcription [4]. The enhancer drives reporter gene activity similar to that of *ex-LacZ* in most tissues (Figs 1 and S1, this study). This enhancer is activated by Da through one or more of 3 E-box sequences, and demonstrably bound by Da in chromatin from wing imaginal discs [4]. The intron 3 enhancer also contains the sequences that respond to Sd/Yki [4], and is associated with Yki protein in vivo according to previously-published ChIP data [27]. Here, we present a further characterization of the *ex* intron 3 enhancer that provides insights into the regulation of ex expression and SWH activity by growth-regulatory pathways in the wing imaginal disc.

## Materials and methods

### *Drosophila* genetics

Fly culture and cross were performed according to standard procedures at 25°C unless otherwise noted. *ex*^*697*^*-LacZ* (gift from Claude Desplan; [28]), *Ex*^*Intron3*^*-GFP* (*Enh*^*Intron3*^*-GFP*; [4]), and *sca*^*D120*^*-LacZ* [29] were used to monitor transcriptional activity of *ex* and to define the proneural regions. Mutant clones were generated by FLP/FRT-mediated mitotic recombination by crossing mutant strains with *[arm-LacZ] FRT80B* (BDSC BL#6341; [30]); *[Ubi-GFP] M(3)67C FRT80B* [31] and *[Ubi-mRFP*.*nls] FRT40A* (BDSC BL#34500). Mutant strains used were *h*^*22*^ *FRT80B* [32], *sens*^*E2*^ *FRT80B* (gift from Hugo Bellen; [33]), *da*^*3*^ *FRT40A* [34]. Heat shocks were performed at 36–48 hr after egg laying (AEL) to induce FLP and animals were dissected at late third instar. For overexpression or knocked-down experiments, the following drivers were used: *en-Gal4 UAS-RFP* (BDSC BL#30557), *nub-Gal4* (BDSC BL#38418) and *dpp*^*40C6*^*-Gal4* [35]. *UAS-da* [4], *UAS-da-da* [4], *UAS-sens* (gift from Hugo Bellen; [33]), *UAS-h* (BDSC BL#36529) transgenes were used to overexpress the corresponding gene products. The *Notch*, *yki* and *sd* were knocked-down using UAS RNAi transgenes, including *UAS-N-RNAi* (BDSC BL#7078; [36]), *UAS-yki-RNAi* (BDSC BL#31965), and *UAS-sd-RNAi* (BDSC BL#29352).

### Immunohistochemistry

Immunostaining was performed as described previously [37]. Confocal imaging was performed using Leica SP2 and Zeiss LSM 880 microscopy. Primary antibodies used were anti-Sens (guinea pig, a gift from H. Bellen); beta-galactosidase (mouse, DSHB#40-1a); GFP (rat, NACALAI TESQUE# GF090R). Species-matched Cy2-, Cy3- and Cy5-conjugated secondary antibodies were from Jackson ImmunoResearch.

### Enhancer dissection

Subfragments derived from Ex^Intron3^ were obtained by PCR amplification using primers with Bam HI-Xho I or Bgl II-Xho I sites and cloned into the pH-Stinger vector. The putative transcription factor binding sites were predicted using JASPAR database [38] or rVista [39]. Constructs carrying mutated E-box sites or Sd-binding sites were generated by PCR mutagenesis using the QuikChange Site-Directed Mutagenesis kit (Stratagene). For each construct, a minimum of three independent transgenic lines was analyzed for GFP activity. The expression patterns of each construct were reproducible between different transgenic lines because the pH-Stinger vector contains insulator sequences to eliminate position effects [40].

## Results

### Deletion analysis of the *Ex*^*Intron3*^ enhancer

The *ex* intron 3 enhancer (Ex^Intron3^; described as Enh^Intron3^ in our previous paper[4]) was first recognized as a 1.5 kb segment regulating transcription in a pattern similar to the *ex-LacZ* enhancer trap and which is conserved in other *Drosophila* species (Fig 1A, 1C and 1D; [4]). In leg, antennal and haltere imaginal discs, *Ex*^*Intron3*^*–GFP* faithfully recapitulated *ex-lacZ* expression (S1 Fig). However, some differences may exist. Although *Ex*^*Intron3*^*–GFP* and *ex-LacZ* expression are very similar in the wing imaginal disc, there is less *Ex*^*Intron3*^*–GFP* in the wing pouch than seen for *ex-LacZ* (Fig 1C and 1D, S1A–S1D Fig). Intriguingly, Ex^Intron3^–GFP expression reduced faster than ex-LacZ in the wing pouch at early third instar stage (S1C and S1D Fig). This could be because *ex-LacZ* is also influenced by other enhancers elsewhere in the *ex* gene (or other genes), or it could be that Lac-Z is more stable than GFP so the *ex-LacZ* pattern is partly reflecting the earlier transcription pattern. In addition, in the eye disc, *ex-LacZ* is expressed in a gentle anterior-posterior gradient anterior to the morphogenetic furrow, where *Ex*^*Intron3*^*–GFP* is expressed very little (S1E and S1F Fig). Several regions of the enhancer are highly conserved among multiple *Drosophila* species (Fig 1A).

To dissect the basis for *ex* transcriptional regulation further, deletion constructs of Ex^Intron3^ were generated and analyzed (Fig 1B). The original 1.5kb region was divided into 6 sections, ABCDEF. Elements A, D, and E in particular showed conservation in the distantly related species *D*. *virilis* and *D*. *grimshawi* (Fig 1A).

When either element A or F was deleted, the sequences remaining in ABCDE or BCDEF retained the similar expression pattern as full-length Ex^Intron3^ (Fig 1D–1F). Without both elements, however, in the BCDE reporter new transcription appeared in the proneural regions that flank the anterior wing margin (Fig 1G). These are the regions where sensory structures of the anterior wing margin develop [43,44]. This suggests that both elements A and F contain information for blocking *ex* expression in the wing margin proneural cells, and that either A or F is sufficient for this. However, elements A and F did not share sequence similarity. Element F comprises 1/3 of the entire Ex^Intron3^. Region F alone had weak expression in the distal wing hinge and antenna imaginal discs, while it showed no reporter activity in the eye and leg discs (Fig 1I, S1 Fig). The BCDE element did not show major qualitative expression changes in eye-antennal or leg discs (S1 Fig), indicating that *ex* is regulated at the wing margin differently from some other proneural regions such as the morphogenetic furrow of the eye disc. Thus, positive aspects of *ex* enhancer activity appear to map within BCDE sequences.

Little further effect on the pattern of wing disc expression was seen when region E was deleted, but there may be some quantitative differences between BCD-GFP and BCDE-GFP. In BCD-GFP (which was described as Enh^658^ in our previous paper [4]), GFP seemed somewhat decreased in the distal hinge but higher in the wing margin proneural cells (Fig 1H). Overall, though, sequences within the BCD element were sufficient for much of the *ex* transcription pattern (except for repression of the wing margin proneural cells that was redundantly encoded by elements A and F).

It was the D sequences that showed the greatest evolutionary conservation (Fig 1A). However, element D alone failed to drive any GFP expression in the wing disc (S1Q Fig). When we tried subdividing element BCD, element BC-GFP showed similar expression as BCD-GFP, except that BC-GFP also drove expression in the wing pouch (Fig 1J). CD-GFP drove even more expression, almost uniformly throughout the wing disc. Although this expression was largely uniform, it seemed the proneural cells of the anterior wing margin continued to maintain slightly higher levels (Fig 1K). Therefore, sequences in region B and D appeared to be necessary to prevent *ex* transcription in the wing pouch region, excluding the anterior wing margin proneural cells where repression required element A or F

These studies provide an outline for how the pattern of *ex* transcription defined by *ex-LacZ* and by *Ex*^*Intron3*^*–GFP* arises, and of the minimum regulatory inputs that are to be expected. The core of the enhancer as illustrated by CD-GFP drives transcription almost uniformly throughout the wing disc, possibly at a somewhat higher level in proneural cells (Fig 1K). Sequences within element B and D both repress *ex-*transcription in the wing pouch region excluding the proneural cells of the anterior wing margin, whereas sequences A and F redundantly add repression of *ex* in the proneural cells.

### Mutational analysis of transcription factor binding sites in the *Ex*^*Intron3*^ enhancer

**Fig 2:**
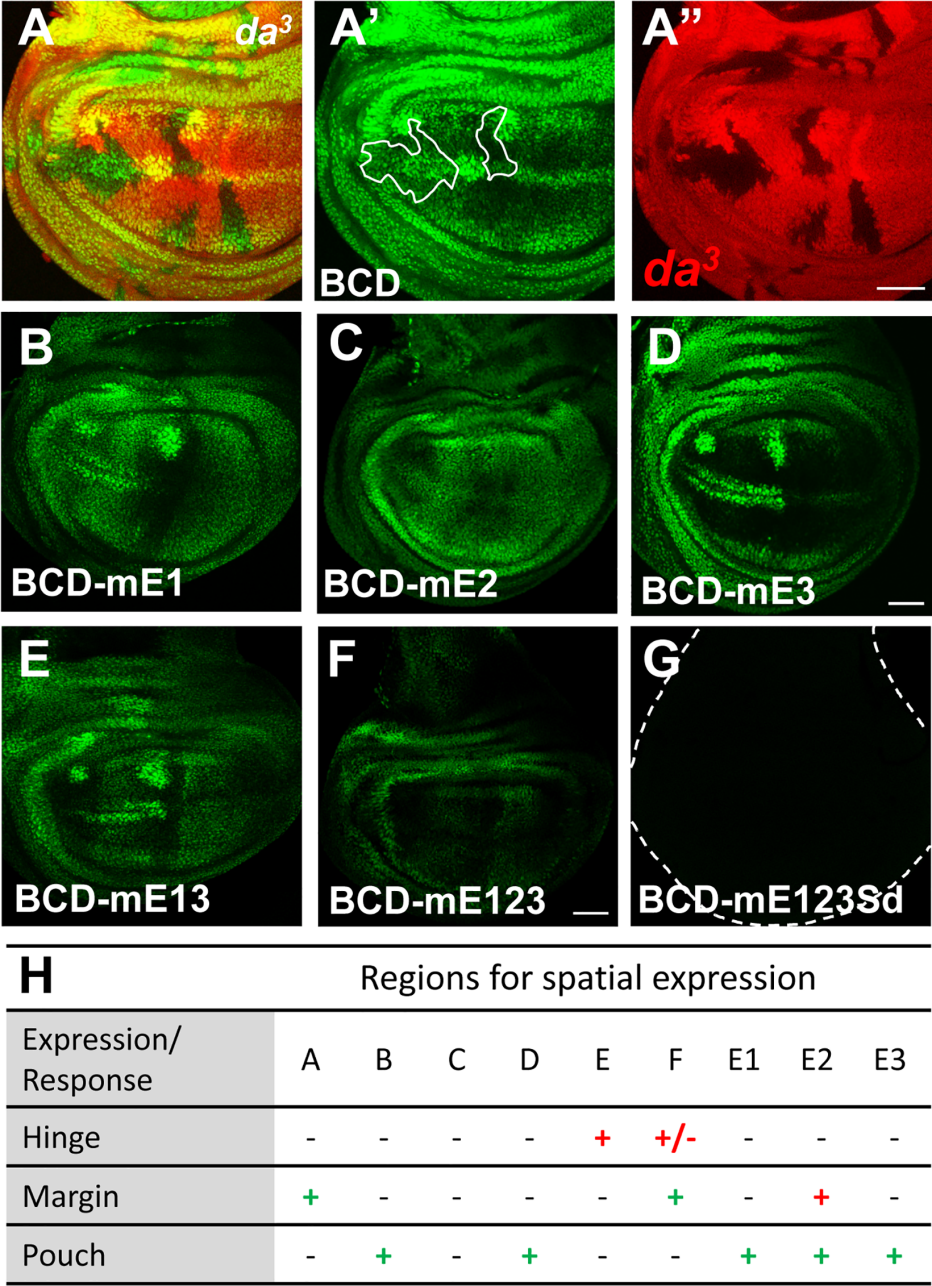
Effect of bHLH protein on Ex^Intron3^ enhancer. (A) Third instar wing imaginal discs containing *da*^*3*^ mutant cells (RFP [red] negative) are visualized for *BCD-GFP* reporter expression (A’, green). Note the decreased levels of *BCD-GFP* in *da* mutant clones within the wing margin proneural region but not hinge. (B-G) GFP reporter expression driven by the indicated genomic sub-fragments in the third instar wing imaginal discs. (H) Summary of regions important for spatial expression. Note that + (red): sufficiency; +/-: partial sufficiency; + (green): requirement; -: no effect. Scale bars, 50 μm.

Because proneural regions are generally defined by bHLH transcription factors, we wondered whether *ex* transcription near the wing margin depended on the Achaete-Scute Complex (AS-C). Since all the AS-C proteins require dimerization with Da to bind DNA, we examined BCD-GFP expression in *da* null clones. BCD-GFP expression was reduced in *da* null mutant clones within the wing margin proneural cells (Fig 2A), consistent with a role for the AS-C in wing margin expression.

AS-C/Da heterodimers bind to DNA through E-box sequences (CANNTG). BCD contains three potential E-box sequences, and these were mutated individually, together, and in combinations (Fig 2, S2A Fig). Mutating all three E-boxes together (in construct BCDmE1,2,3) abolished BCD activity in the anterior wing margin proneural cells (Fig 2F). Whereas E-box 1 and E-box 3 were dispensable, even when mutated together in construct BCDmE1,3 (Fig 2B, 2D and 2E), by contrast E-box 2 was essential for BCD-GFP expression in the wing margin cells (Fig 2C). Because E-box 2 is the only E-box remaining in the BCDmE1,3 construct that drives expression very similar to the parent BCD-GFP element (Fig 2E), it is likely that E-box 2 is also sufficient for *ex* expression at the anterior wing margin proneural cells (at least within the context of the BCD element).

Interestingly, mutations in any of the E-box sequences also appeared to affect expression outside of the proneural cells. Each of the constructs BCDmE1-GFP, BCDmE2-GFP, BCDmE1,3-GFP and BCDmE1,2,3-GFP led to some expression in the whole of the wing pouch, away from the wing margin (Fig 2B, 2C, 2E and 2F).

Since this enhancer was identified initially as the Daughterless-responsive element of the *ex* gene, responsible for Da-dependent Ex over-expression when the *emc* gene was mutated, we also mapped the sequences conferring Da-response, using *nub-Gal4* to over-express Da throughout the wing pouch region. We had found previously that the core enhancer BCD retained the induction by Da over-expression, and that E-boxes were required, because no response was seen to Da over-expression in BCDmE1,2,3 (which was called Enh^658^-mEbox in our previous paper[4]). Here, we report that BCDmE1,3 also failed to respond to Da over-expression (S2C Fig). Thus the response to Da over-expression depended on distinct E-boxes in comparison to *ex* expression in response to AS-C and Da at the anterior wing margin.

**Fig 3:**
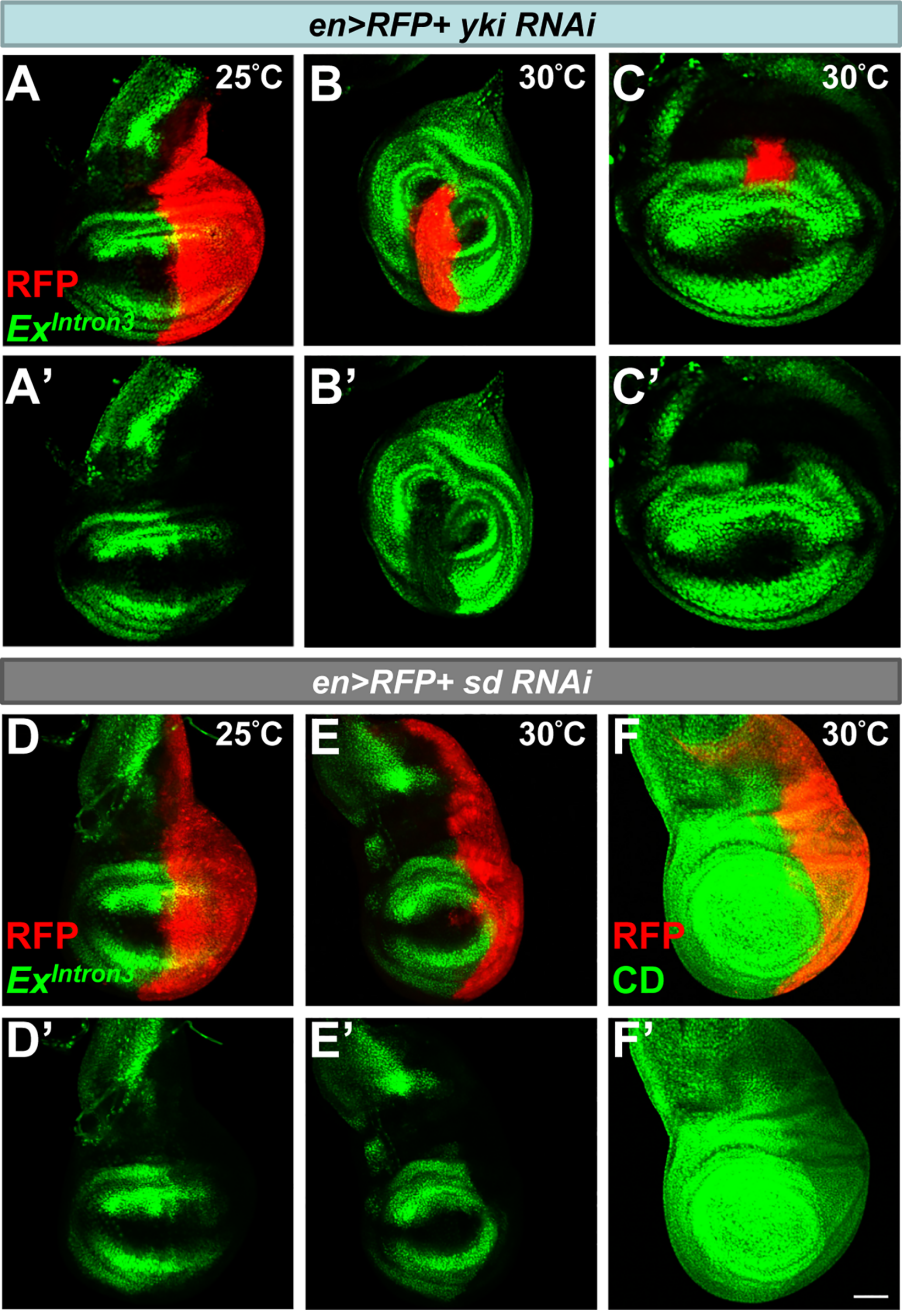
SWH pathway requires Sd/Yki to regulate *ex*. (A) Blockage of the SWH pathway by expressing an RNAi against *yki* (*en>RFP+yki RNAi*) leads to the downregulation of Ex^Intron3^-GFP (green) in the posterior compartment (marked by RFP, red) of wing disc at 25°C. (B-C) Leg and wing discs of *en>RFP+yki RNAi* (red) staining for Ex^Intron3^-GFP (green) at 30°C. Fly cross and culture was performed at 25°C. After 36–48 hr AEL, *en>RFP+yki RNAi* flies were incubated at 30°C until dissection at late third instar. Note the autonomous reduction of GFP was more obvious in flies that were shifted at 30°C. The altered *en* domain (marked by RFP, red) at 30°C suggest that Yki may regulate *en* expression. (D-E) Wing disc of *en>RFP+sd RNAi* (red) staining for Ex^Intron3^-GFP (green) at 25°C and 30°C, respectively. Note the decrease of GFP was more obvious when animals were shifted and incubated at 30°C. (F) Wing disc of *en>RFP+sd RNAi* (red) staining for CD-GFP (green) at 30°C. Note that CD-GFP was decreased in a cell autonomous manner. Scale bar, 50 μm.

It has been reported that SWH activity is sufficient to activate Ex^Intron3^ expression. Over-expression of Sd and Yki was sufficient to activate expression in the wing pouch [4]. To determine whether SWH pathway was required for the normal activity of Ex^Intron3^, Sd/Yki activity was knocked down by double-stranded RNAs, expressed under the control of *en-Gal4*. En-Gal4 drives gene expression in the posterior compartment, including the posterior wing hinge region where Ex^Intron3^-GFP is active. GFP expression of Ex^Intron3^ was cell autonomously decreased by knock-down of either Yki or Sd in the wing or leg discs (Fig 3A–3E, S3A and S3B Fig), suggesting that Sd/Yki is necessary for endogenous expression of *ex* in the proximal part of the wing disc. When Sd and Yki were knocked-down in the background of CD-GFP, which is expressed throughout the wing disc, expression in the wing pouch region was greatly decreased (Fig 3F, S3C–S3F Fig), indicating that Sd and Yki are active throughout the wing disc and needed to activate *ex* transcription at all locations. Note that the sequences that normally reduce wing pouch expression map to region B of the enhancer, however, as described above.

Twelve putative binding sites of Scalloped (Sd) were predicted across the entire Ex^Intron3^ element (Fig 1A), however, only one of these lies within the CD element. We obtained inconsistent results from mutating this site. When this putative Sd binding sequence (in region D) was disrupted in the CD-GFP construct, expression in the pouch was partially reduced, leading to an expression pattern similar to BC-GFP where the anterior wing margin expression is clearly higher than that of the surrounding wing pouch (S2A and S2E Fig). When this sequence was disrupted in BCDmE123-GFP, expression was completely lost (Fig 2F–2G, S2A, S2D and S2E Fig). Taken together, these studies show that Sd and Yki are required for Ex^Intron3^ expression, and probably at least in part through this Sd site, although it is uncertain whether other sequences could also be involved.

### Regulation of the *Ex*^*Intron3*^ enhancer by candidate genes

**Fig 4:**
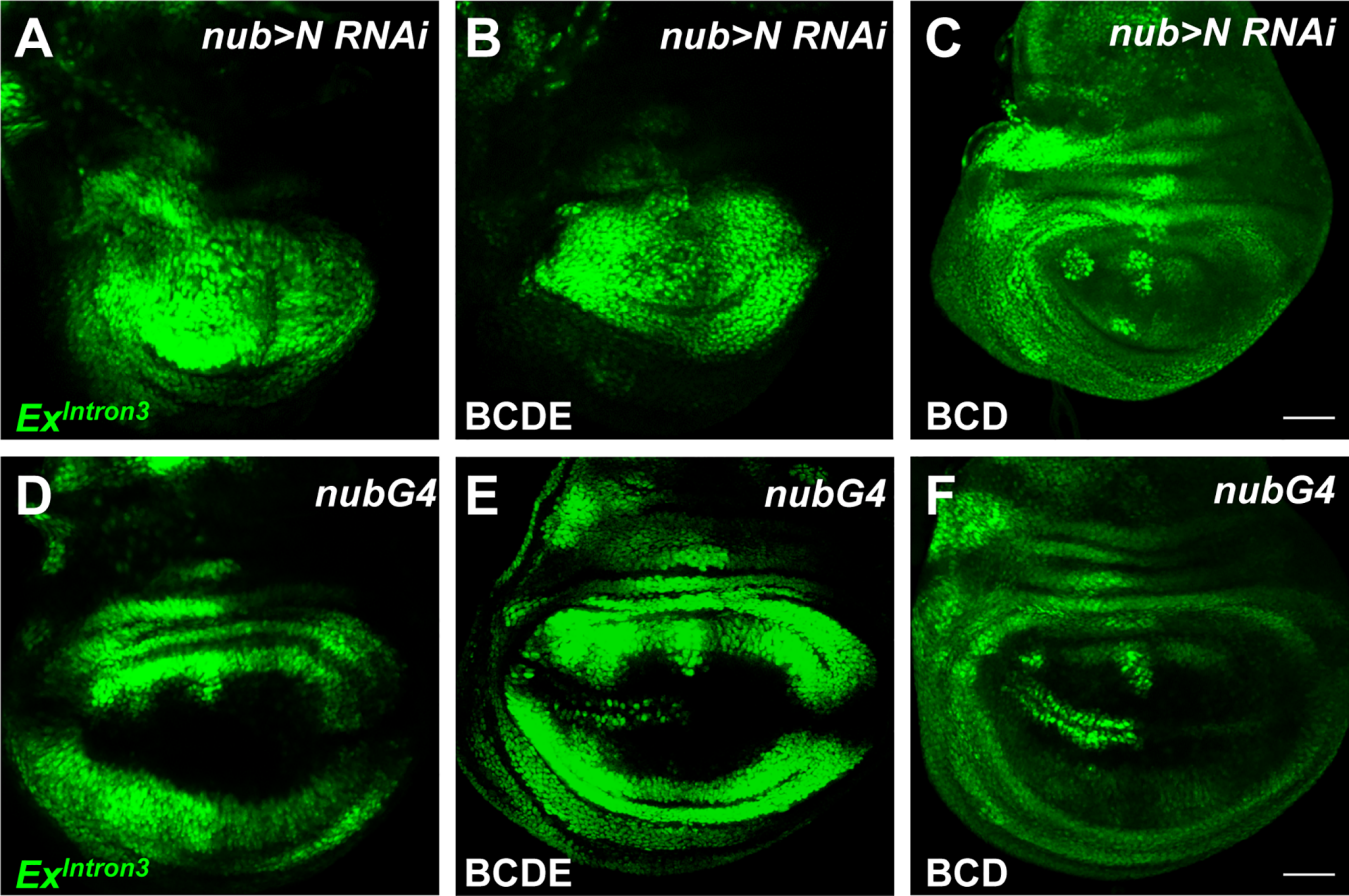
Element E is required for Notch-dependent repression. (A) Blockage of the Notch pathway by expressing an RNAi against Notch in wing pouch (*nub>N RNAi*) leads to the upregulation of *Ex*^*Intron3*^*-GFP* (green). (B) GFP expression under the control of element BCDE in *nub> N RNAi* wing discs. Note the de-repression of BCDE-GFP in response to knockdown of Notch. (C) Wing disc of *nub>N RNAi* staining for BCD-GFP expression. Note that the BCD-GFP did not respond to downregulation of Notch pathway. (D-F) Wing imaginal discs of *nub-Gal4* for Ex^Intron3^-GFP (D), BCDE-GFP (E) and BCD-GFP (F), respectively. Scale bars, 50 μm.

Because the CD element is expressed throughout the wing disc in response to Sd/Yki, and the addition of region B is necessary for repressing *ex* transcription in the distal wing and pouch, our studies question whether SWH signaling and Sd/yki are responsible for the proximal-distal gradient of *ex* transcription. It has been proposed that N contributes to proximo-distal patterning of *ex* transcription, by inducing Vg within the wing pouch [45]. Vg could compete with Yki for Sd and so diminish Sd/Yki activity. Consistent with this model, N knock-down in the wing pouch de-repressed *ex* transcription [3]. Using N knock-down, we found that N was required for wing pouch repression of the full-length enhancer, and of the BCDE construct, but that the BCD construct remained repressed in the wing pouch even when N was knocked-down (Fig 4). These results suggest that N represses *ex* transcription in the wing pouch through region E of the enhancer. However, N knockdown abolished expression of ex at the wing margin in BCD-GFP, indicating that N activity contributed to the AS-C/Da-dependent *ex* expression that occurs there.

**Fig 5:**
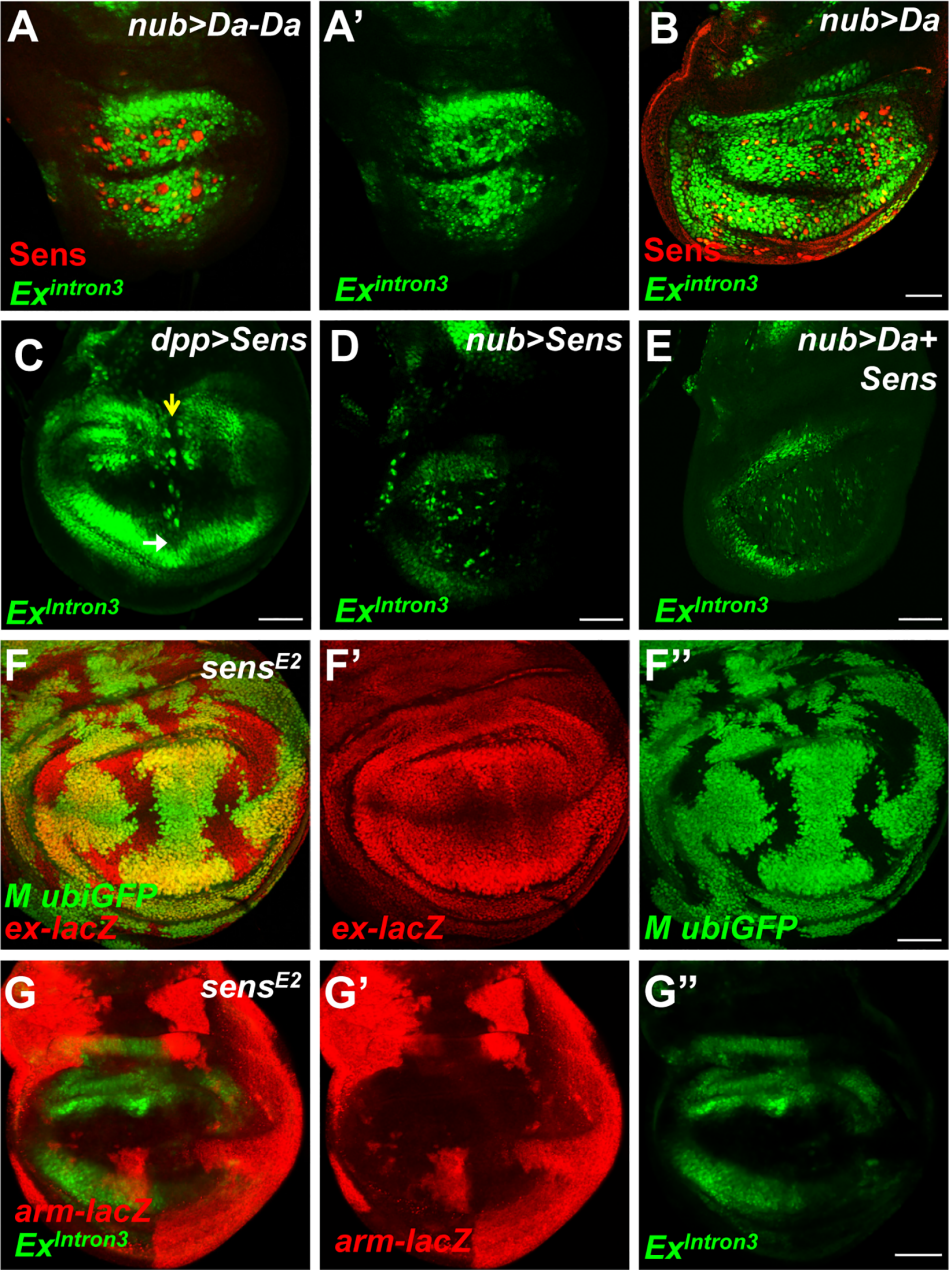
Sens inhibits Da-mediated ex expression in the wing pouch. (A, B) Wing disc overexpressing Da homodimer (A, *nub>Da-Da*) or monomer (B, *nub>Da*) in the wing pouch and staining for *Ex*^*Intron3*^*-GFP* (green) expression. Note the upregulation of Ex^Intron3^-GFP caused by Da homodimer is attenuated in Sens-expressing cells (red). (C) Wing imaginal disc of *dpp>Sens* staining for *Ex*^*Intron3*^*-GFP* expression. Note the inhibition of Ex^Intron3^-GFP by high levels of Sens in the dorsal proximal wing (indicated by yellow arrow), while ventral wing is barely affected (white arrow). Some enlarged cells with GFP-positive staining are seen around the Dpp domain. (D) Wing disc of *nub>Sens* staining for *Ex*^*Intron3*^*-GFP* expression. (E) Wing disc of *nub>Da+Sens* staining for *Ex*^*Intron3*^*-GFP* expression. Note the GFP expression is slightly disrupted as seen in *nub>Sens* disc. (F-F”) A late third instar wing disc containing *sens* mutant cells (GFP negative) is visualized for *ex-LacZ* expression (red, F’). (G-G”) Third instar wing imaginal disc of *sens* mutant cells (*arm-lacZ* negative) visualized for *Ex*^*Intron3*^*-GFP* expression (green, G”). Note no elevation of *ex-LacZ* and Ex^Intron3^-GFP is detected in *sens* mutant clones. Scale bars, 50 μm.

The specific identity of pathways repressing of *ex* transcription in the wing pouch and at the anterior wing margin was explored further using mutations in candidate repressor genes. Senseless (Sens) was a candidate repressor because low levels of Sens have been reported to act as a repressor *in vivo* [46]. The mammalian homolog of Sens, the growth factor independence 1 (Gfi-1) gene, encodes a zinc finger transcriptional repressor that represses several cell cycle components and proapoptotic Bcl2 family member Bax [47–51]. Sens is expressed in the wing margin cells [33], and a chromatin immunoprecipitation (ChIP)-chip database of early embryogenesis [52] reported the association of Sens protein with the intron-3 enhancer region of *ex*. Interestingly, when a linked Da homodimer was overexpressed in the wing pouch, *Ex*^*Intron3*^ expression was induced as seen previously when Da monomer was expressed [4], except for sporadic patches of cells that also turned on Sens expression (Fig 5A and 5B). Thus there was a correlation between Sens expression and silencing *Ex*^*Intron3*^ expression, as also occurs at the normal anterior wing margin.

When Sens was over-expressed using the *dpp-GAL4* driver, *Ex*^*Intron3*^ expression was reduced where the Dpp expression domain crossed the proximal wing region dorsally, but this was not observed ventrally (Fig 5C). When Sens was over-expressed throughout the wing pouch using *nub-Gal4*, *Ex*^*Intron3*^ expression was slightly reduced (Fig 5D). *Ex*^*Intron3*^ expression was unchanged when Da was co-expressed with Sens, clearly demonstrating the capacity of Sens to repress expression (Fig 5E).

To examine *ex* expression in the absence of Sens, *ex-LacZ* and *Ex*^*Intron3*^ *–*GFP were examined in *sens* null mutant clones, but there was little effect; expression remained low in the wing pouch and wing margin (Fig 5F–5G). These results suggest that Sens over-expression may be able to suppress *ex* expression but is not normally required to do so in the wing pouch or at the anterior wing margin.

**Fig 6:**
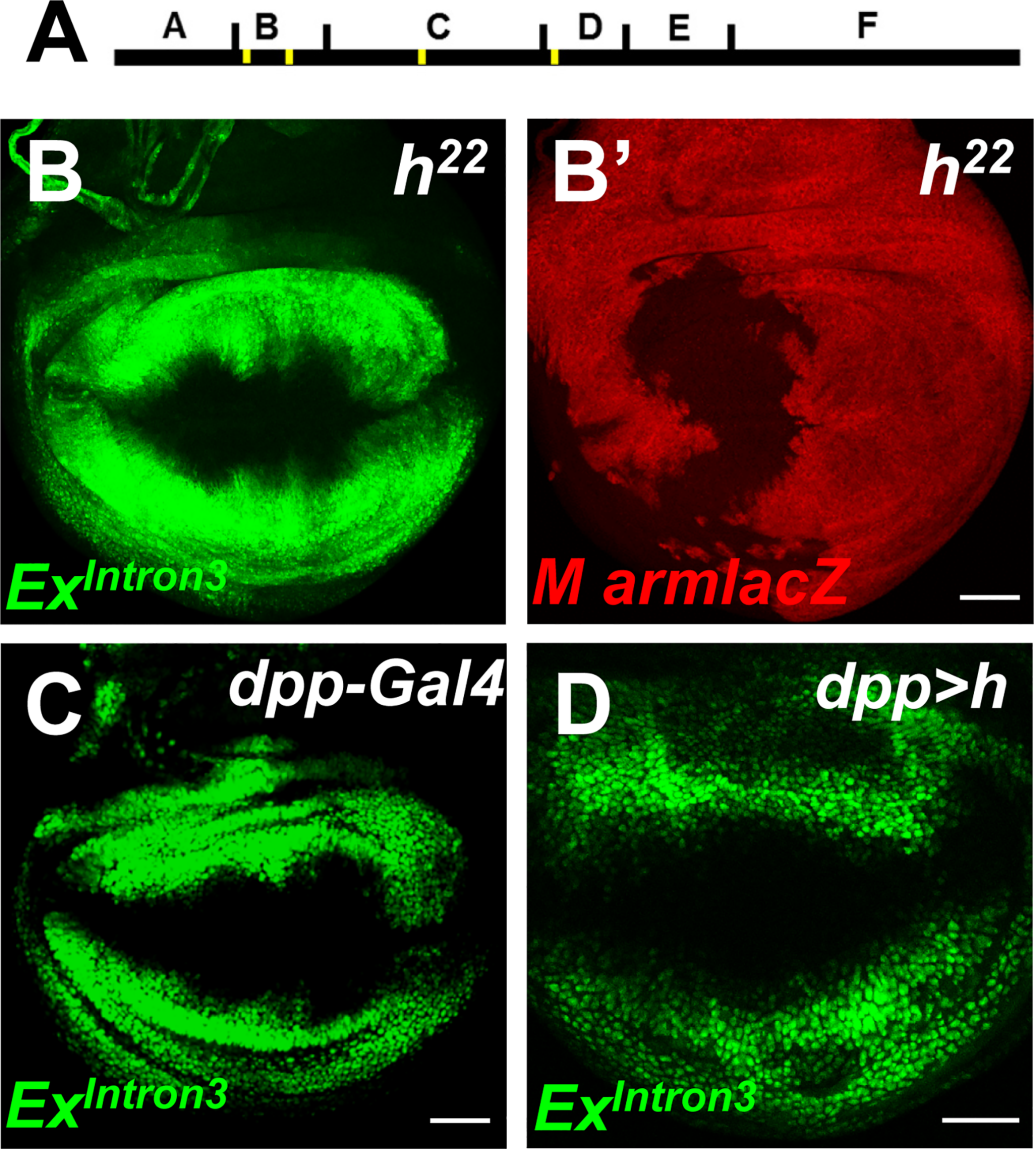
*ex* expression is independent of *hairy*. (A) Schematic representation of the ABCDEF enhancer. Yellow bars denote putative Hairy binding sites. (B-B’) Clones of homozygous *hairy* mutant cells are labeled by the lack of LacZ expression (red). Note Ex^Intron3^-GFP (green) is not changed in *h* clones. (C, D) Expression of Ex^Intron3^-GFP in wing discs of *dpp-GAL4* (C) and *dpp>h* (D) flies. Note Ex^Intron3^-GFP is not affected in Dpp domain when *h* expression is manipulated. Scale bars, 50 μm.

Intriguingly, several putative Hairy binding sites were predicted within intron-3 enhancer (Fig 6A). Proteins of the Hairy/Enhancer of split/Deadpan (HES) family act as a classical transcriptional repressors by binding to C-box (CACNNG) sequence [53–57]. Although Da expression is independent of *hairy* [58], Hairy antagonizes neurogenesis via binding to the enhancer of another proneural gene *achaetae* [59]. To investigate ex expression in the absence of *hairy* (*h*) activity we used clones homozygous for *h* null alleles. *Ex*^*Intron3*^*-GFP* was unchanged (Fig 6B). This suggests that *h* is not required for repressing *ex* in the wing margin proneural region. Also, overexpression of *h* was not sufficient to repress Ex^Intron3^-GFP (Fig 6D). Previous studies have shown that *ex-lacZ* is unaffected in homozygous clones which removes the entire Enhancer of split (E(spl)) complex and *deadpan* (*dpn*)[3]. Together, these findings suggest that none of HES repressor proteins can account for the repression of *ex* in the wing although we cannot completely rule out the possibility that *h*, E(spl) and *dpn* might function redundantly.

**Fig 7:**
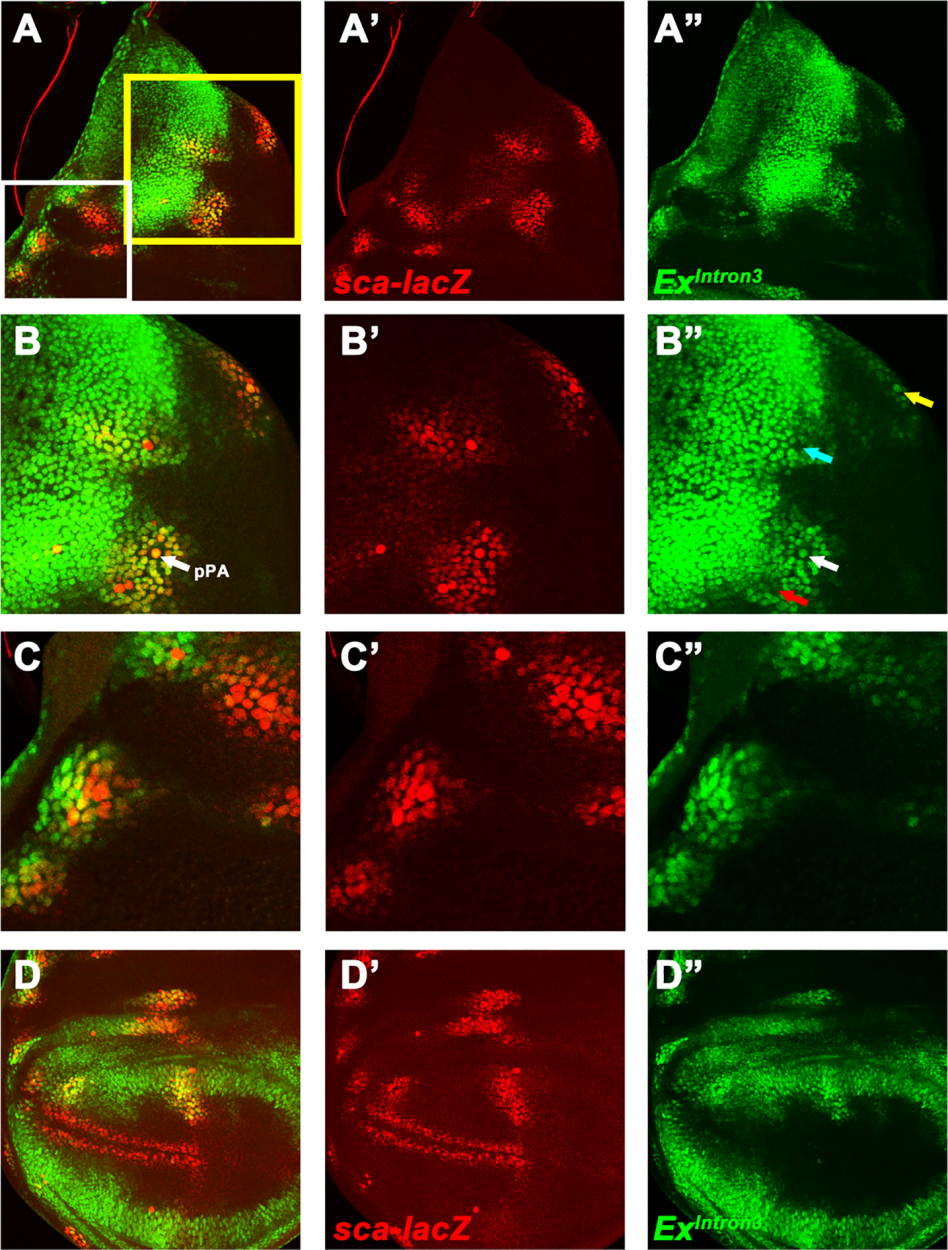
*ex* expression in the proneural regions. Expression patterns of Ex^Intron3^-GFP (green) and *sca-LacZ* (red) in notum (A-A”) and wing pouch (D-D”). (B-C) Higher magnification of the yellow box (B-B”) and white box (C-C”) in A. Note the anterior scutellar SOP showed higher GFP activity than nearby cells (yellow arrow), the posterior post-alar SOP expressed the GFP at levels similar to other proneural cells (white arrow), whereas the posterior dorsocentral SOP and anterior post-alar SOPs showed reduced levels of GFP activity (blue and red arrows, respectively).

Because Da protein is elevated in all proneural regions yet examined, yet at the anterior wing margin this does not seem to elevate ex transcription, we examined some other proneural regions for comparison. As already mentioned, *Ex*^*Intron3*^–GFP is not active in eye imaginal discs. We double labeled *Ex*^*Intron3*^ reporter with *sca-lacZ* to visualize proneural region in the notum and wing (Fig 7). Like the anterior wing margin, *Ex*^*Intron3*^ reporter was absent from some SOP cells of notum, such as anterior post-alar SOP. However, *Ex*^*Intron3*^ reporter was expressed in many other proneural regions as defined by *sca-LacZ* expression [29]. For instance, *Ex*^*Intron3*^ reporter was expressed more highly in the posterior post-alar SOP than in surrounding cells (Fig 7B). Intriguingly, hypomorphic *ex* mutants have extra post-alar bristles in the adult thorax [4], suggesting that *ex* is required for some aspects of proneural cluster development. The variety of expression levels at distinct proneural regions implies spatially-regulated positive and negative inputs acting on *Ex*^*Intron3*^.

## Discussion

The development, differentiation and growth of cells and tissues require precisely regulated patterns of gene expression, depending on the time and spatial location of its activation, and its crosstalk with other signaling pathways. Here we used the *Drosophila* wing disc as a model system to investigate how *ex* transcription is regulated. Ex is important as a negative growth regulator that acts through the SWH pathway [1,5–7,60–62]. It restricts wing size in normal development, so that mutants have larger, ‘expanded’ wings [63], and can also be induced to block growth of cells with developmental perturbations, including those with *emc* mutations that over-express Da [4]. In normal development, *ex-LacZ* is expressed most highly in the hinge region that surrounds the wing pouch, and expression decreases in a gradient until none is detected at the wing margin (Fig 1C). Unlike expression patterns of *ex-LacZ*, a relatively ubiquitous distribution of Ex protein is seen in the wing imaginal discs [15, 64–65]. The discrepancy between *ex* reporter and Ex protein might be due to the post-translational control of Ex protein stability [66]. Alternatively, Ex protein might be strongly influenced by expression patterns in earlier developmental stages (S1A Fig). Regardless, because *ex* is itself a transcriptional target of the SWH pathway, acting through Sd and Yki, *ex-LacZ* is extensively used as a transcriptional readout of SWH signaling activity, if not of Ex protein distribution. This transcription pattern of *ex* could be interpreted to indicate a proximal-to-distal gradient of Sd/Yki activity, which would be consistent with certain models of wing growth regulation that propose that SWH activity represses growth in central regions of the disc whereas Yki activity is higher in proximal regions [67]. Another mechanism predicted to repress growth in central regions of the wing disc is activation of Notch signaling there. Notch signaling induces expression of Vg, a protein that binds Sd in competition with Yki and is therefore predicted to reduce Yki activity (and *ex* transcription) in central regions of the wing pouch [3]. Since we have identified an *ex* enhancer whose activity reflects the *ex-LacZ* reporter in the endogenous gene, we can explore this enhancer to understand how these and other signals are integrated at the *ex* locus.

**Fig 8:**
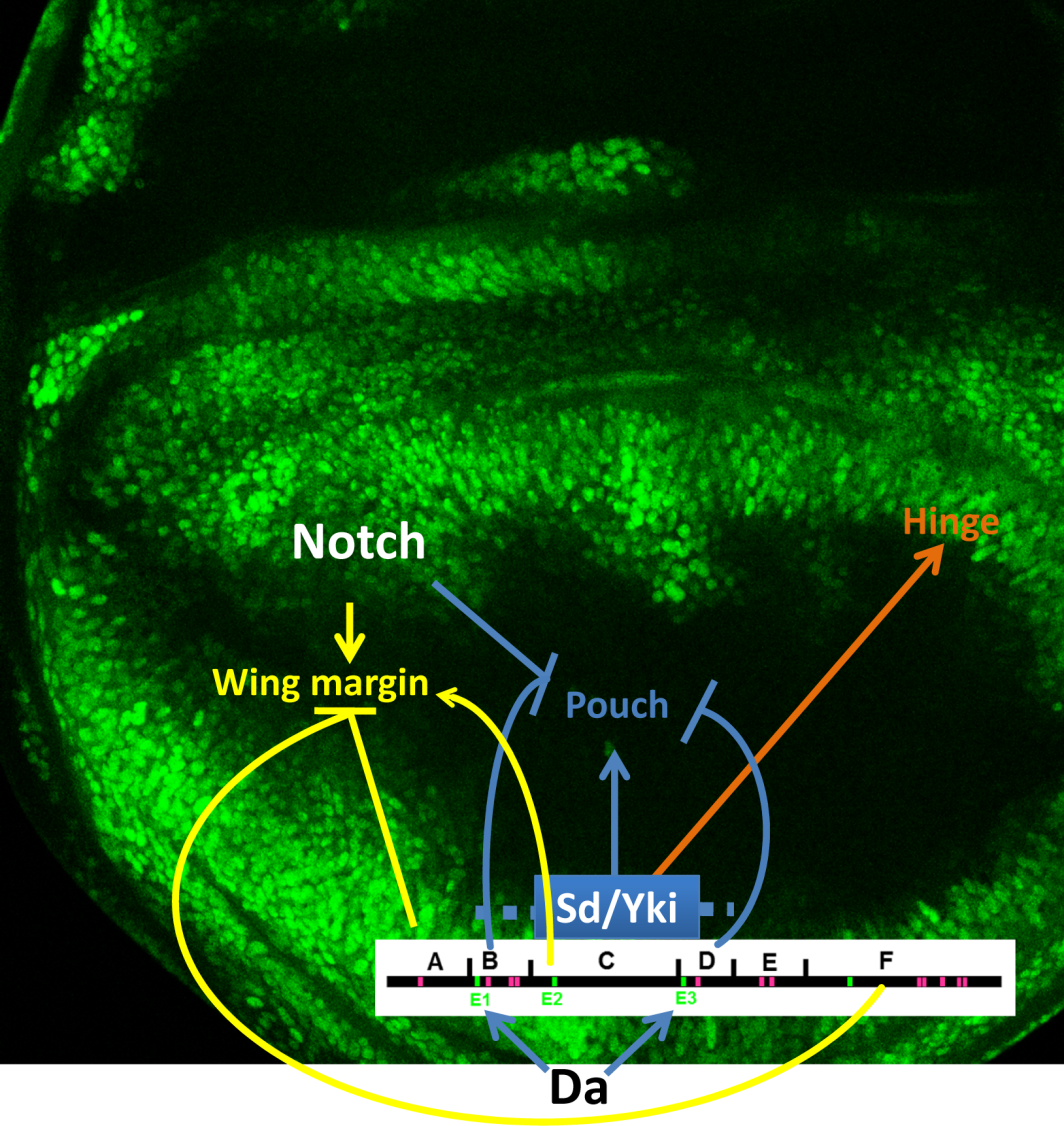
Model of differential regulation on *ex*. In the wing margin proneural region (high SWH activity; low Yki activity), ex is negatively regulated by inputs acting through elements A and F. The E-box site #2 (E2) is required for expression in the wing margin proneural cells while Da acts through E1 and E3 to regulate *ex* transcription. Sd/Yki regulates *ex* transcription through element BCD in wing pouch and hinge. Notch acting through element E and other inputs acting through elements B and C repress ex transcription in wing pouch. Note the image of wing disc is adopted from Fig 7D”.

A deletion analysis outlines several major features of *ex* regulation (Figs 1 and 8). First, the core of the enhancer, centered around the ‘C’ element and probably including contributions from the flanking ‘B’ and ‘D’ elements, is active throughout the wing disc. All this activity depends on both Sd and Yki, suggesting that Yki is active throughout the wing disc. This is consistent with the previous finding that *yki* is required for growth throughout the wing disc [17,18]. ChIP-seq analysis revealed that Yki binding peaks over *Ex*^*Intron3*^ [27]. Although Sd binding has not been mapped yet in *Drosophila*, it is likely that Sd binding strongly correlates with Yki, as seen for the mammalian YAP1 and TEAD1 proteins [68]. Expression of the Sd/Yki-dependent BC and CD elements provided no evidence of a proximal-distal gradient of Sd/Yki activity. Instead, the overall reduction of *ex* expression in the central, wing pouch region of the wing disc requires both the B and D regions together, suggesting that two other inputs are both required to achieve this silencing. It does not seem that either of these silencing inputs corresponds to Notch signaling, because although we confirm that Notch is required to silence *ex* enhancer activity in the wing pouch, it is not required to silence the BCD element.

An unexpected aspect of *ex* transcription regulation is independent regulation of *ex* enhancer activity in the proneural cells of the anterior wing margin. Activity here was encoded by the BC and CD elements, and depended on an E-box within C (E-box #2). Surprisingly, given the normal role of Notch signaling in lateral inhibition to prevent neural differentiation, the wing margin activity depended positively on N signaling. Anterior wing margin activity was dependent on Da, the obligate heterodimer partner of all proneural genes, indicating that it is possibly encoded by proneural genes of the AS-C. This regulation was unexpected because there was no evidence for *ex-LacZ* or *Ex*^*intron3*^*-GFP* reporter activity at the anterior wing margin. This is because such expression was silenced by either one of two flanking elements, A and F, acting redundantly. We tested the hypothesis that Sens, which is expressed in the anterior wing margin proneural region and can act as a transcriptional repressor, was responsible for blocking the activity. Although Sens was sufficient to inhibit *Ex*^*intron3*^*-GFP* activity at some ectopic locations, it was not required for *Ex*^*intron3*^*-GFP* repression at any location, including at the anterior wing margin.

We originally identified the *ex* enhancer through its role in mediating *ex* transcription in response to elevated Da activity [4]. The Da response was mapped to E-boxes #1 and #3 within elements B and D, respectively. Since Da can bind to DNA as a homodimer, and as no other Class I or Class II bHLH gene is known to be widely expressed in the wing pouch, it is possible that Da homodimers activate this site. The E1 site within element B matches the consensus CACCTG sequence that is preferred by Da-Da homodimers and by homologous E-protein homodimers in mammals [69]. Endogenous Da protein levels are normally elevated at the anterior wing margin, because Emc levels are reduced there, so it is interesting that *Ex*^*intron3*^ is activated at the anterior wing margin through distinct E-boxes that differ in sequence from E2 site. One possibility is that it is heterodimers of Da with proneural proteins encoded by AS-C that activates these E-boxes at the anterior wing margin, since we previously showed that ectopically-expressed AS-C proteins can activate the intact enhancer [4]. The consensus E-box site for Sc/Da has been described as GCAGC/GTG and the 5’ flanking base G is essential for the binding specificity of Sc [70]. The E2 site matches both the core sequence and the 5’ flanking base (S2 Fig). In part this could explain why *emc* loss is detrimental for proliferating progenitor cells in the rest of the wing disc and leads to their *ex*-dependent loss during growth, in contrast to the proneural cells that normally tolerate elevated Da levels, if Da proteins contribute to distinct dimers in these two situations.

Our analysis shows that multiple regulatory inputs are integrated by the *Ex*^*intron3*^ enhancer (Fig 8). Although we confirm previous conclusions that Sd/Yki and Notch signaling regulate *ex* transcription, our studies indicate that the full enhancer, and hence the widely-used *ex-LacZ* line, are not straightforward reporters of Yki activity. Instead the smallest sequence that responds to Sd and Yki is active almost uniformly throughout the wing disc, suggesting that this may be the pattern of Yki activity. This may be a useful reporter for SWH activity in future studies. The final *ex-LacZ* pattern is strongly influenced by additional sequences that silence transcriptional activity in the wing pouch and at the anterior wing margin, in the latter case apparently preventing *ex* transcription in response to proneural gene activity while preserving sensitivity to ectopic Da expression.

## Supporting information

S1 Fig Related to Fig 1. Third instar eye, leg and haltere imaginal discs of ex-LacZ and the indicated genomic sub-fragments. L2: second instar larval stage; eL3: early third instar larval stage.
(TIF)

S2 Fig Related to Fig 2. (A) Schematic representation of three E-box sites (underlined) in BCD region and Sd site (underlined) in D region. The mutated sequences are shown in red. (B-C) *BCD-GFP* and *BCDmE1*,*3-GFP* expression in *nub>Da* wing discs, respectively. Note that the *BCDmE1*,*3* does not respond to Da overexpression. (D) The same disc shown in 2G. The DAPI staining is used to mark the wing area since there is no GFP activity of *BCDmE123Sd-GFP*. (E) CDmSd-GFP expression in late third instar wing disc.
(TIF)

S3 Fig Related to Fig 3. (A) Peripordial membrane of *en>RFP+yki RNAi* (red) staining for *Ex*^*Intron3*^*-GFP* (green) at 25°C. Note that Ex^Intron3^-GFP was decreased in peripordial membrane of wing. (B-C) Leg discs of *en>RFP+sd RNAi* (red) staining for *Ex*^*Intron3*^*-GFP* and *CD-GFP* at 30°C, respectively. (D) Wing discs of *en>RFP+yki RNAi* (red) staining for *CD-GFP* (green) at 30°C. Note that CD-GFP was decreased in the posterior compartment while some yki knock-down cells close to the proneural region retain a residual GFP expression. (E-F) Wing discs of *nub-Gal4* and *nub>yki RNAi* staining for *CD-GFP* (green) at 30°C, respectively. Compared to *nub-Gal4* control, CD-GFP is decreased in *nub>yki RNAi*.
(TIF)
