# Supplementary figures and images for: Spatial regulation of expanded transcription in the Drosophila wing imaginal disc
Source: PLoS One. 2018 Jul 31;13(7):e0201317. doi: 10.1371/journal.pone.0201317 (PMC6067730; doi:10.1371/journal.pone.0201317)

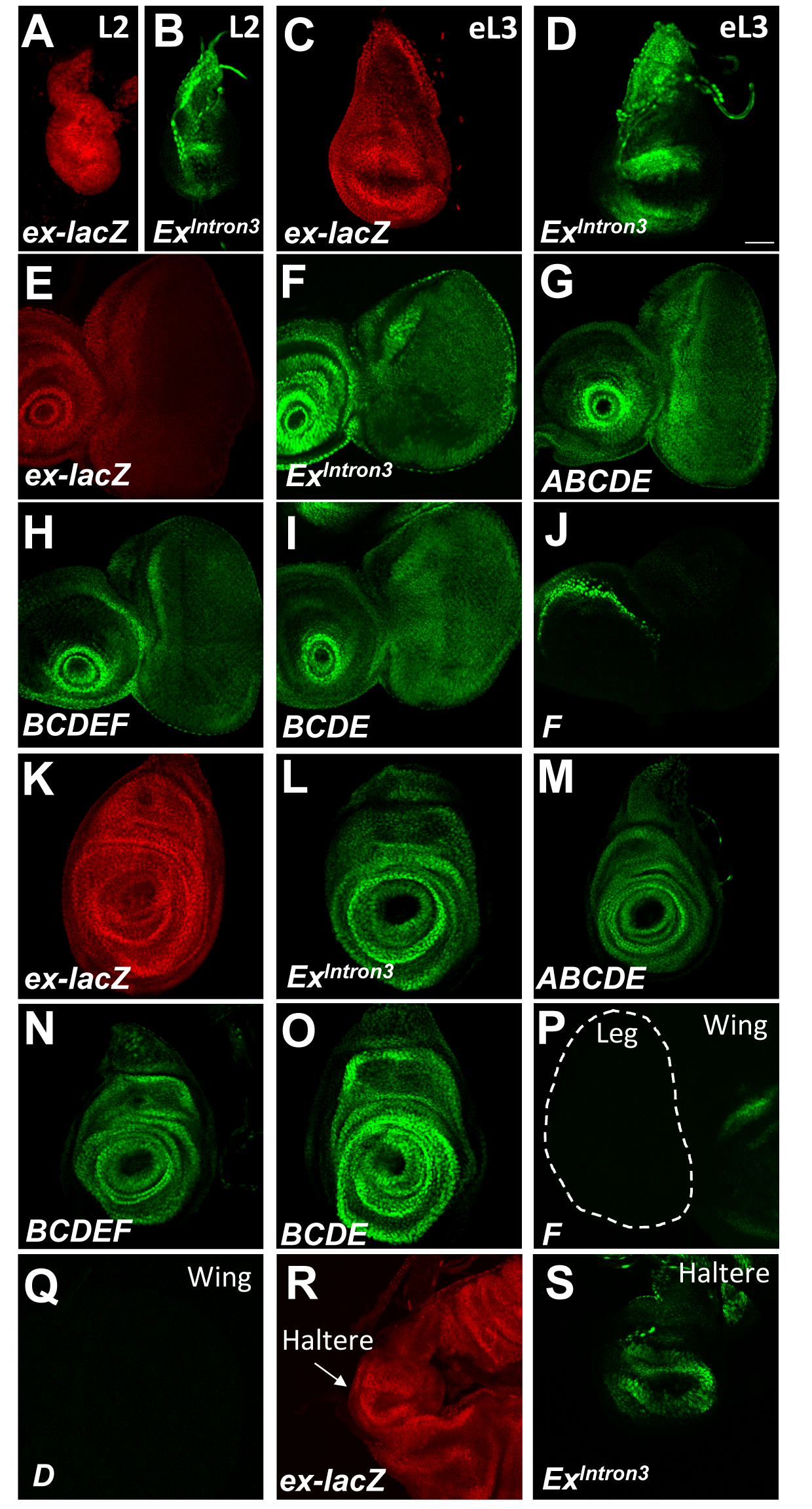

Supplement: S1 Fig — Third instar eye, leg and haltere imaginal discs of ex-LacZ and the indicated genomic sub-fragments. L2: second instar larval stage; eL3: early third instar larval stage. (TIF) [file pone.0201317.s001.tif]

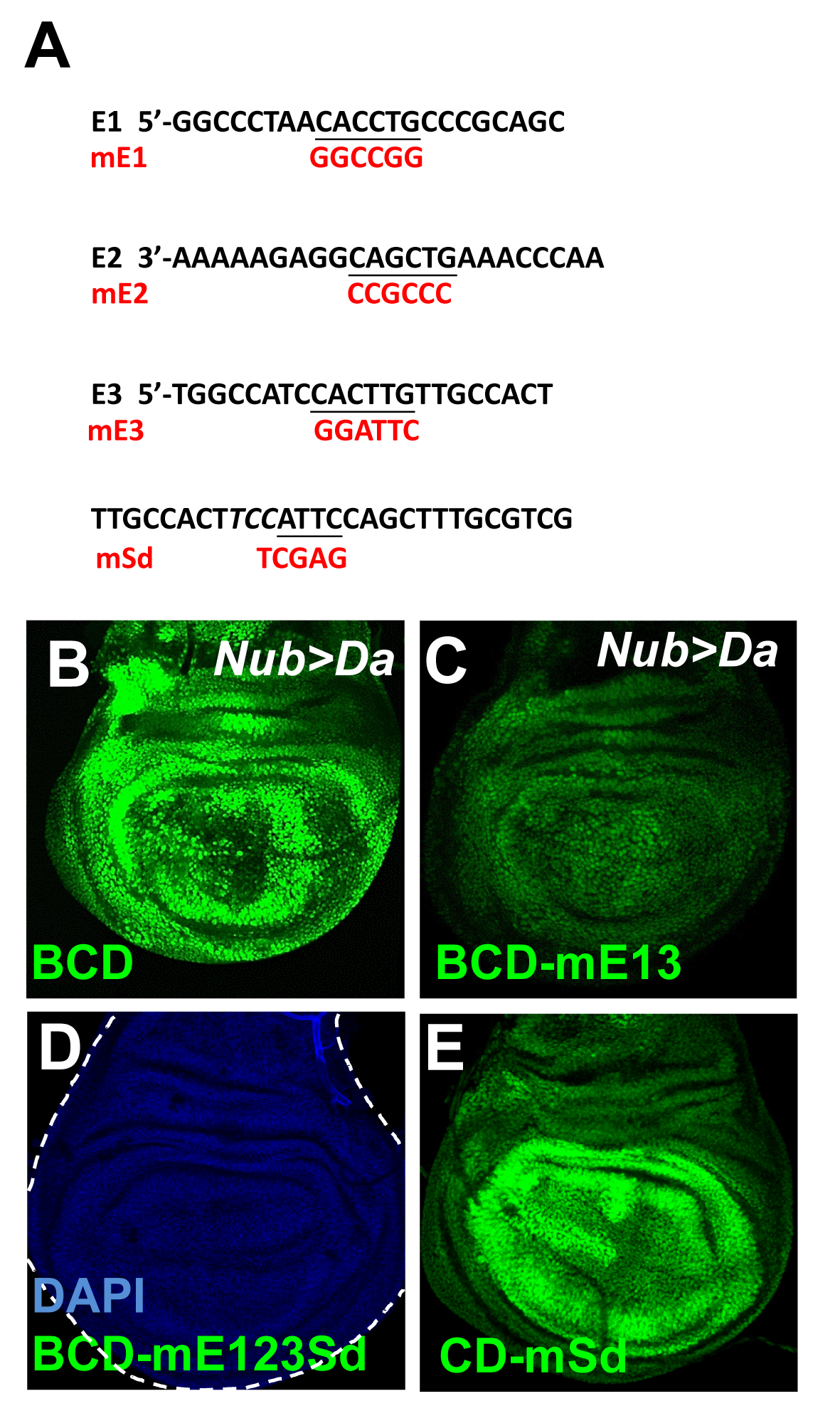

Supplement: S2 Fig — (A) Schematic representation of three E-box sites (underlined) in BCD region and Sd site (underlined) in D region. The mutated sequences are shown in red. (B-C) BCD-GFP and BCDmE1,3-GFP expression in nub>Da wing discs, respectively. Note that the BCDmE1,3 does not respond to Da overexpression. (D) The same disc shown in 2G. The DAPI staining is used to mark the wing area since there is no GFP activity of BCDmE123Sd-GFP. (E) CDmSd-GFP expression in late third instar wing disc. (TIF) [file pone.0201317.s002.tif]

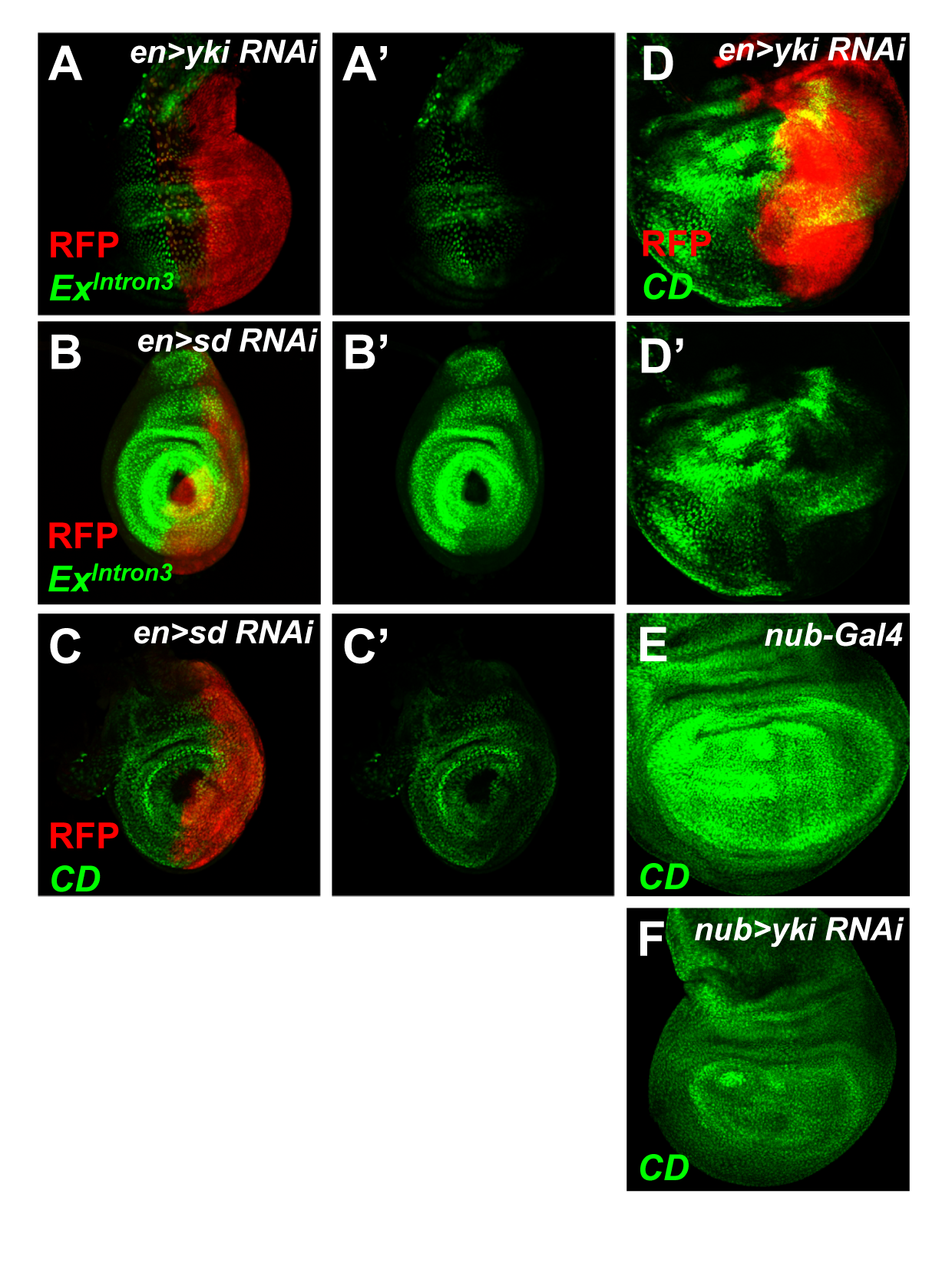

Supplement: S3 Fig — (A) Peripordial membrane of en>RFP+yki RNAi (red) staining for ExIntron3-GFP (green) at 25°C. Note that ExIntron3-GFP was decreased in peripordial membrane of wing. (B-C) Leg discs of en>RFP+sd RNAi (red) staining for ExIntron3-GFP and CD-GFP at 30°C, respectively. (D) Wing discs of en>RFP+yki RNAi (red) staining for CD-GFP (green) at 30°C. Note that CD-GFP was decreased in the posterior compartment while some yki knock-down cells close to the proneural region retain a residual GFP expression. (E-F) Wing discs of nub-Gal4 and nub>yki RNAi staining for CD-GFP (green) at 30°C, respectively. Compared to nub-Gal4 control, CD-GFP is decreased in nub>yki RNAi. (TIF) [file pone.0201317.s003.tif]
